# Biocompatibility of a Ti-Rich Medium-Entropy Alloy with Glioblastoma Astrocytoma Cells

**DOI:** 10.3390/ijms232314552

**Published:** 2022-11-22

**Authors:** Wen-Fu Ho, Ka-Kin Wong, Mei-Hwa Lee, James L. Thomas, Ya-Chun Chang, Shih-Ching Wu, Hsueh-Chuan Hsu, Hung-Yin Lin

**Affiliations:** 1 Department of Chemical and Materials Engineering, National University of Kaohsiung, Kaohsiung 81148, Taiwan; 2 Department of Materials Science and Engineering, I-Shou University, Kaohsiung 84001, Taiwan; 3 Department of Physics and Astronomy, University of New Mexico, Albuquerque, NM 87131, USA; 4 Department of Dental Technology and Materials Science, Central Taiwan University of Science and Technology, Taichung 40601, Taiwan

**Keywords:** Ti-rich medium-entropy alloy, cell viability, glioblastoma astrocytoma cell

## Abstract

Titanium and titanium alloys are widely used in medical devices and implants; thus, the biocompatibility of these metals is of great importance. In this study, glioblastoma astrocytoma cellular responses to Ti_65_-Zr_18_-Nb_16_-Mo_1_ (Ti65M, metastable medium-entropy alloy), Ti-13Nb-7Sn-4Mo (TNSM, titanium alloy), and commercially pure titanium (CP-Ti) were studied. Several physical parameters (crystal phase structure, surface roughness and hardness) of the titanium alloys were measured, and the correlation with the cellular viability was investigated. Finally, the relative protein expression in cellular proliferation pathways was measured and compared with mRNA expression assessed with quantitative real-time reverse transcription polymerase chain reaction assay (qRT-PCR).

## 1. Introduction

Titanium (Ti) alloys with high specific strength and high corrosion resistance are excellent candidates for use in biomedical applications involving implantation [1]. Ti-6Al-4V ELI (TC4 ELI) is used in various fields, including as a biomedical material [2]. However, it suffers from poor mechanical strength, high elastic modulus, and bio-toxicity [3,4]. Many researchers have recently developed metastable β-Ti alloys that contain no toxic elements and have low elastic moduli [5,6]. Unfortunately, the metastability of β-Ti alloys causes them to have low strength [7]. In our previous work, a metastable β-structured Ti-13Nb-7Sn-4Mo (TNSM) with a very low elastic modulus (53 GPa) was developed, but its yield strength (790 MPa) was much lower than that of TC4 ELI (1336 MPa) [8].

High-entropy alloys (HEAs) can have excellent mechanical strength due to lattice distortion [9]. In recent years, several Ti-Zr-Nb-Ta-Hf-Mo HEAs and medium-entropy alloys (MEAs) without toxic elements have been developed for use in biomedicine [10,11,12]. Equiatomic Ti-Zr-Nb-Ta-Mo (TZNTM) HEAs, developed by Wang et al., exhibit high compressive yield strength (1330 MPa) and corrosion resistance which is comparable to that of TC4 [13]. In a biocompatibility test, the viability of osteoblast cells on the surface of TZNTM HEAs was significantly higher than that on commercially pure titanium (CP-Ti). However, the elastic modulus (153 GPa) of equiatomic TZNTM HEA greatly exceeded that of cortical bone (~16 GPa), leading to the stress shielding effect [14]. Hori et al. found that cell viability and growth capacity on Ti_1z_._4_-Zr_1_._4_-Nb_0_._6_-Ta_0_._6_-Mo_0_._6_ HEAs significantly exceeded those on CP-Ti in an osteoblast assay [15]. The excellent biocompatibility of Ti_1_._4_-Zr_1_._4_-Nb_0_._6_-Ta_0_._6_-Mo_0_._6_ is attributable to the relatively high proportions of Ti and Zr elements in the alloy [15].

In our earlier work, three non-equiatomic Ti-rich Ti-Zr-Nb-Mo MEAs (Ti_50_-Zr_25_-Nb_15_-Mo_10_, Ti_58_-Zr_23_-Nb_12_-Mo_7_, and Ti_65_-Zr_20_-Nb_10_-Mo_5_) were developed for biomedical purposes [16]. Increasing the Ti content in Ti-Zr-Nb-Mo MEAs reduces lattice distortion, and thus the elastic modulus. Ti_65_-Zr_20_-Nb_10_-Mo_5_ exhibits a high modulus of resilience (11 MJ/m^3^) and the lowest elastic modulus (86 GPa), but its elastic modulus is still much higher than that of cortical bone. We also synthesized Ti_65_-Zr_20_-Nb_14_-Mo_1_ and Ti_65_-Zr_18_-Nb_16_-Mo_1_ (Ti65M), which are Ti-rich metastable MEAs [17]. That the phase structure of Ti65M is metastable was proved by transmission electron microscopy with selected area electron diffraction (SAED-TEM). Ti65M has an ultra-low elastic modulus (61 GPa) due to its metastable state, which is significantly lower than those of Ti-64 (110 GPa) [2] and TZNTM HEA (153 GPa) [4]. It also has a high yield strength (1118 MPa) due to the lattice distortion, and its yield strength/elastic modulus ratio (×1000) is as high as 18.3.

In addition to having good mechanical properties, the biocompatibility of potential biomedical materials is critical. Therefore, Ti65M for use in biomedical applications must undergo testing for biocompatibility. U87 MG is often used to evaluate the cytotoxicity of biomaterials [18,19,20]. Raeisi et al. investigated the ability of calcined CoO/Co_3_O_4_ nanoparticles on U87 MG using the MTT method. They proved that U87 MG could be used to assess the cytotoxicity of cobalt oxide nanoparticles [19]. Compared with normal cells, U87 MG cancerous cells can passively accumulate more metal nanoparticles [20], which can enhance their usefulness in evaluating the biocompatibility of materials. In this investigation, three titanium-containing samples (Ti65M, TNSM, and CP-Ti) (including their phase structures, surface morphology and roughness, and hydrophilicity) were characterized using X-ray diffraction (XRD), atomic force microscopy (AFM), and surface hardness analysis. The materials were “extracted” using PBS, and this extract was tested for toxicity to U-87 MG glioblastoma astrocytoma cells. Cells were also incubated with these alloys to evaluate their biocompatibility (e.g., viability) and to study relative protein expression and mRNA expression in the receptor tyrosine kinase (*RTK*) pathway. This study showed that the Ti65M MEA, with a lower Ti concentration, was more biocompatible than the other materials containing higher Ti content.

## 2. Results and Discussion

**Figure 1:**
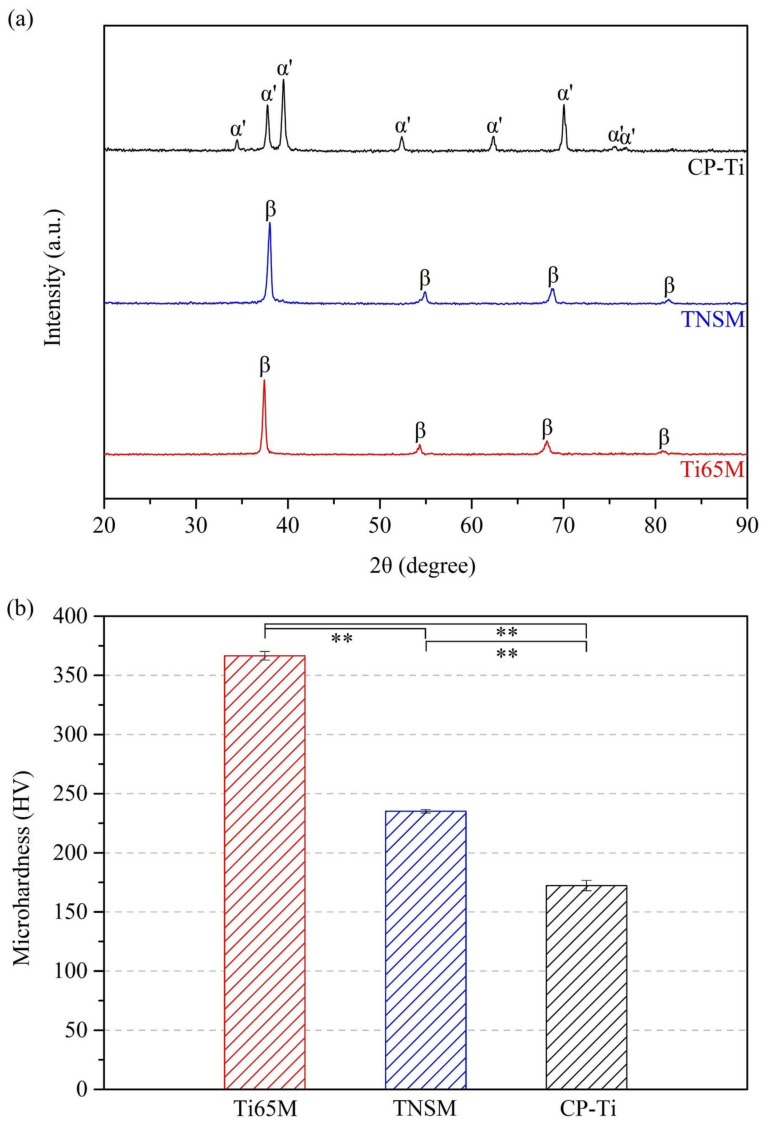
(**a**) XRD patterns and (**b**) hardness values of Ti65M, TNSM, and CP-Ti (** *p* < 0.005).

Figure 1a presents the XRD patterns of Ti65M, TNSM, and CP-Ti. Ti65M and TNSM have a single β structure, consistent with previous findings [17,21]. Although the Ti content of Ti65M is lower than 50 wt.%, Ti65M still has a single β structure, as a result of the high-entropy effect. When compared with TNSM, the β peaks of Ti65M were shifted to lower angles, reflecting the fact that Ti65M is an MEA with a high degree of lattice distortion. In contrast, CP-Ti has a single α’ structure. Figure 1b shows the hardness of Ti65M, TNSM, and CP-Ti. Due to lattice distortion [16], the MEA Ti65M exhibits excellent hardness, much higher than that of TNSM (a Ti alloy), or that of CP-Ti.

**Figure 2:**
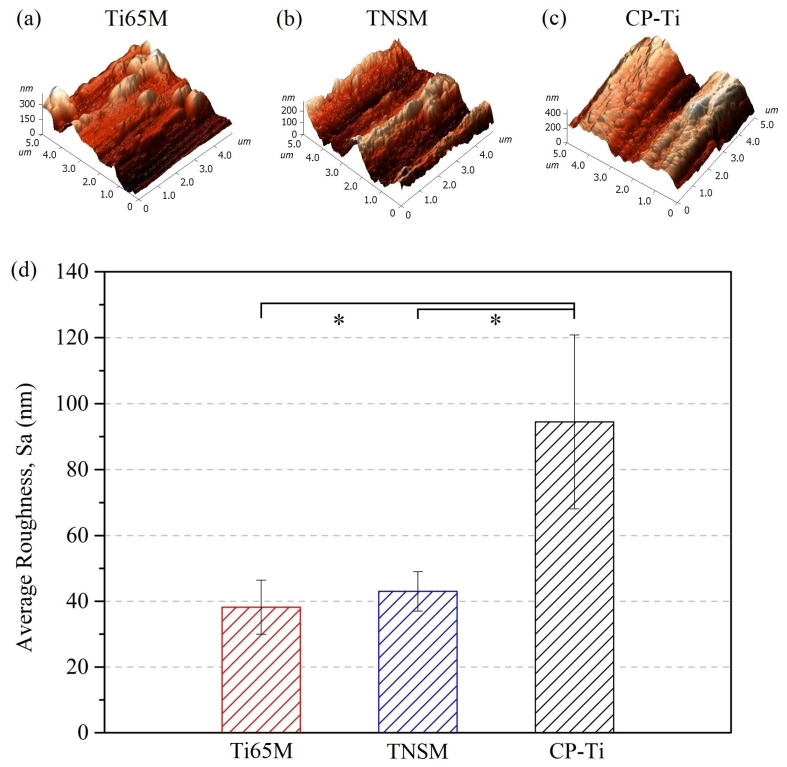
Three-dimensional surface AFM topographies of (**a**) Ti65M, (**b**) TNSM, (**c**) CP-Ti, and (**d**) the comparison of their average roughness (Sa) (* *p* < 0.05).

Figure 2a–c displays the AFM three-dimensional surface topographic images and average roughness (Sa) of Ti65M, TNSM, and CP-Ti. The topographic images of Ti65M and TNSM were similar to each other, but differed significantly from those of CP-Ti. CP-Ti had the highest Sa roughness value (94 ± 26 nm), which is considerably higher than those of TNSM (43 ± 6 nm) and Ti65M (38 ± 8 nm). The high roughness of an alloy surface favors the adhesion and differentiation of cells; furthermore, cell contact with rough surfaces may induce autophagy, leading to cellular differentiation [22]. However, there does appear to be an average roughness threshold (between 0.08 and 1 μm) above which cell proliferation becomes difficult [23]. In the present study, Ti65M had the highest hardness value (366.5 ± 3.6 HV) and the lowest average roughness (38 ± 8 nm). CP-Ti has the lowest hardness (172.3 ± 4.4 HV) and the highest average roughness (94 ± 26 nm). Interestingly, although the hardness of Ti65M was significantly higher than that of TNSM, the alloys did not differ in average roughness, indicating that the roughness of the alloy surface is not determined solely by hardness.

**Figure 3:**
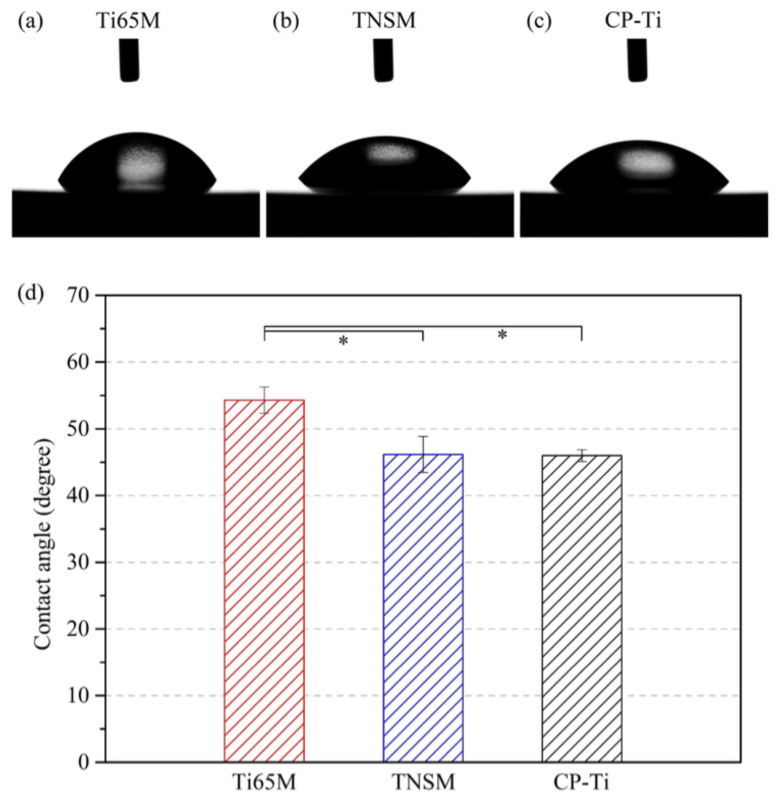
Photographs of the water droplets on (**a**) Ti65M, (**b**) TNSM, and (**c**) CP-Ti, and (**d**) their contact angles (* *p* < 0.05).

The wettability (hydrophilicity) of an alloy can be evaluated by measuring the contact angle of water droplets resting on its surface. A lower contact angle corresponds to greater wettability: a material is considered to be hydrophilic when the contact angle is less than 90° and hydrophobic when the contact angle exceeds 90° [24]. Figure 3a–c show photographs and contact angles of water droplets on Ti65M, TNSM, and CP-Ti. All three materials are hydrophilic; CP-Ti had the lowest contact angle (46.0 ± 0.9°), which was slightly lower than those of TNSM (46.2 ± 2.7°) and Ti65M (54.3 ± 2.0°). Many studies have demonstrated connections between surface roughness and hydrophilicity, and cellular adhesion, growth, autophagy, and differentiation [25,26,27,28]. The mechanisms are not yet clear. Protein adhesion may be important, and has been found to be maximal on surfaces with contact angles from 20° to 40° [29]. Surface roughness and hydrophilicity characterization are important in any cell adhesion study.

The contact angle on an alloy surface is influenced by surface roughness [30,31] and surface free energy [32]. In the Wenzel model, increasing the roughness of a hydrophilic surface increases its contact area with a water droplet, increasing the effective free energy of the interface between the alloy surface and the water, ultimately increasing the contact angle [33]. Notably, CP-Ti had both the highest roughness and the lowest contact angle. This suggests that the intrinsic hydrophilicity of CP-Ti is even higher than the contact angle measurement reveals.

**Figure 4:**
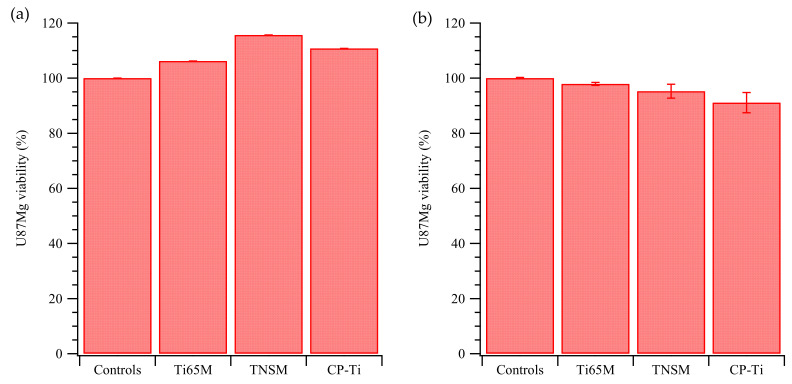
Viability of U87-MG cells with (**a**) extract or (**b**) Ti65M MEA, TNSM, and CP-Ti, normalized to cells on TCPS.

Many researchers have noted that surfaces with high corrosion resistance improve cell viability [34,35,36]. Significantly, the corrosion resistance of β-phase Ti-alloys is generally believed to exceed those of Ti-alloys with other phase structures (α’, α”, α + β, or β) [3,37], due to elements in β-phase alloys that have high corrosion resistance (such as Zr, Nb, Mo, and Ta) [38]. Furthermore, the corrosion resistance of Ti-rich HEAs is often superior to that of traditional Ti-alloys and CP-Ti [10,11,12], because the amounts of alloying elements with high corrosion resistance in HEAs/MEAs are greater than those of traditional Ti-alloys. Therefore, it is reasonable to deduce that the corrosion resistance of Ti65M is superior to those of TNSM and CP-Ti. Further investigation of the corrosion resistance of Ti65M and TNSM will be reported in future studies. Figure 4 depicts the viabilities of U-87 MG on Ti65-M2 MEA, TNSM, and CP-Ti. Biocompatibility was tested in two different ways, following ISO 10993-1. First, the toxicity of any elements, compounds, or nanoparticles that could be extracted from the Ti alloys with a PBS wash was studied. Figure 4a plots the viability of U-87 MG with extracts of Ti65-M2 MEA, TNSM, and CP-Ti; all viabilities exceeded 90%. Some indirect cytotoxicity tests in L929 cells on novel Ti-Mo-Mn alloys for biomedical applications were investigated, showing high biocompatibility [39].

Secondly, we studied cell growth on Ti substrates. The U-87 MG cells (1 × 10^4^ cells) were seeded onto the Ti-containing samples (1.2 cm in diameter) and incubated for 24 h. The U-87 MG cells were then trypsinized, gently washed, and replated into a new 24-well plate and grown for an additional 24 h prior to MTT tests. The U-87 MG cells exhibited 90–80% viability compared with the controls, but decreased as the Ti content increased, as shown in Figure 4b. Cell viabilities on both Ti65M and TNSM, which have β structures, were considerably higher than that on CP-Ti, which has an α’ structure. However, the differential viability on Ti65M and TNSM substrates shows that phase structure alone is insufficient to explain viability. As shown in Table 1, the high hardness of Ti65M improves the wear resistance of its surface and prevent the accelerated release of metal ions in the human body. Combined with the low elastic modulus of Ti65M, the alloy can be applied to brain nerve electrodes, mitigating the mechanical damage caused by the electrode micro-movement to the brain tissue [40].

**Figure 5:**
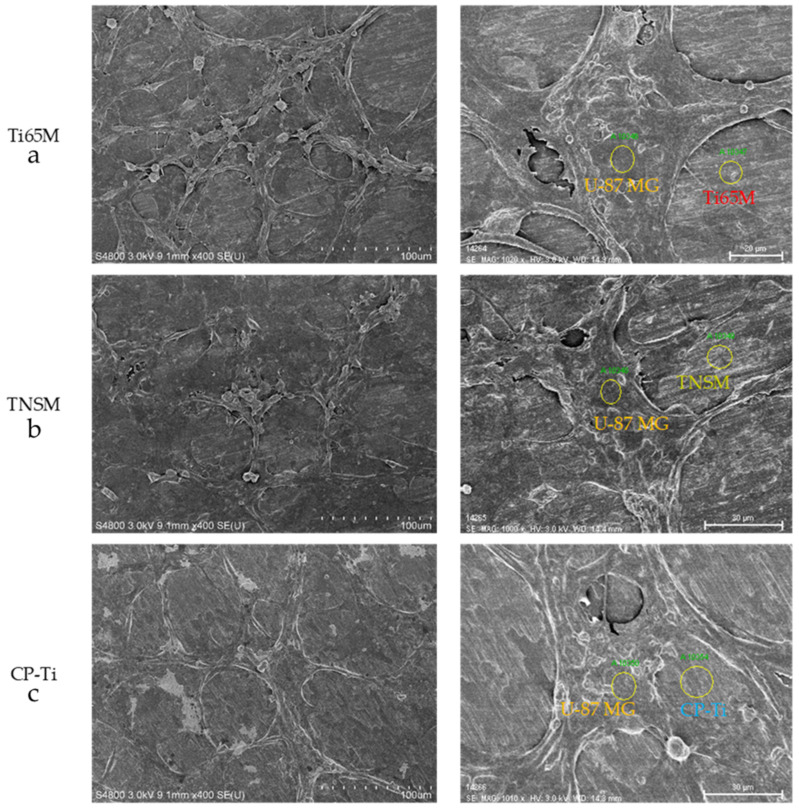
SEM images of U-87 MG cells, which were incubated with (**a**) Ti65M MEA, (**b**) TNSM, and (**c**) CP-Ti for one day.

Similarly treated U-87 MG cells were studied using optical microscopy and nuclear staining with DAPI, as shown in Figure S1. Cells grown exclusively on TCPS were included as controls. Dendrites and axons of U-87 MG were clearly seen for all samples, but their numbers were dramatically lower on Ti65M, TNSM to CP-Ti. Only a few dendrites of U-87 MG were observed on the TNSM and CP-Ti samples. SEM images of U-87 MG cells attached on Ti65-M2 MEA, TNSM, and CP-Ti are shown in Figure 5. The marked places were employed to identify the Ti-containing samples and U-87 MG cells. There are more dendrites and axons of U-87 MG on the Ti65M than other Ti-alloys, presumably because the composition of Ti65M contains the highest amounts of Zr and Nb elements [1]. The SEM images in Figure 5 are consistent with the optical and fluorescence microscopy in Figure S1. Although containing the lowest concentration of Ti, Ti65M contains the highest concentration of Zr and Nb, which could improve the biocompatibility of the alloy. Zr and Nb are highly biocompatible, exhibiting low cytotoxicity in vitro, excellent biocompatibility in vivo, no evidence of mutagenicity or carcinogenicity, good resistance to corrosion, and osteocompatibility equaling to or exceeding that of Ti [41].

Comparing the Ti65M MEA, TNSM, and CP-Ti substrates, contact angle and hardness (in Table 1) had no significant effects on cell morphology and cell viability. However, the number of dendrites and axons of U-87MG cells on the three Ti-based materials were significantly different (Figure S1). We suggest that the alloy’s corrosion resistance may affect the number of dendrites and axons of U-87MG cells. U-87MG cells showed the highest viability and number of dendrites and axons on Ti65M, which may be related to the highest content of Zr and Nb in Ti65M [1].

**Figure 6:**
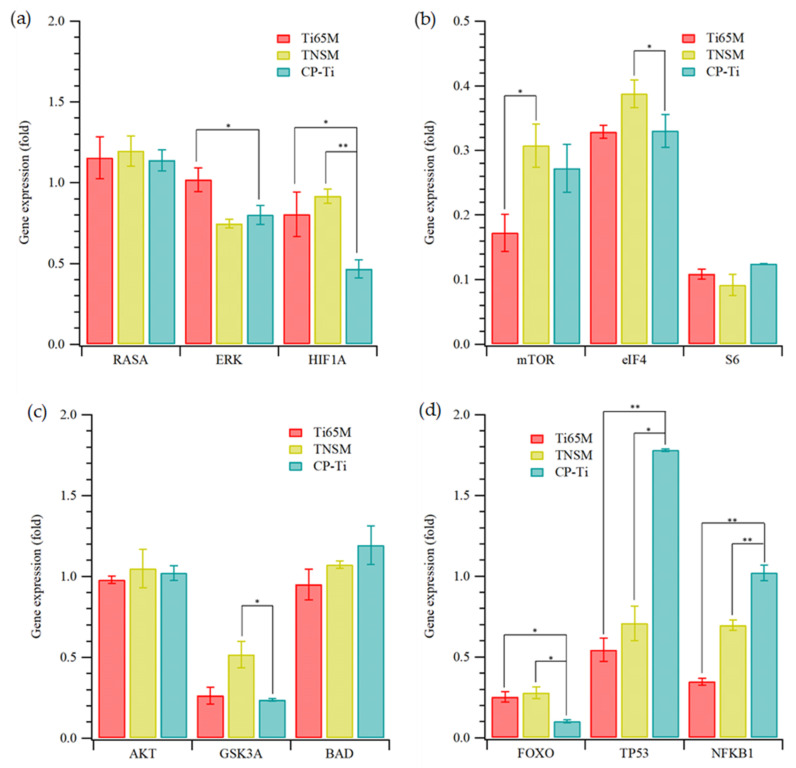
Expression levels (relative to cells on TCPS) of key factors (**a**) RAS, ERK, and HIFα, (**b**) mTOR, eIF4, and S6, (**c**) AKT, GSK3, and ABD, (**d**) FOXO, p53, and NFκβ in pathways of U-87 MG cells on Ti65M MEA, TNSM, and CP-Ti. Statistical significance (*) was set at a *p*-value of less 0.05, and highly significant (**) as *p* < 0.005.

Finally, gene expression between materials and cells [42,43] for growth factor pathways was studied. The simplified pathway of U-87 MG growth factor receptor activation is described in Scheme S1. Figure 6 shows the gene expression for these related pathways. As shown in Figure 6a, the expression of ERK was reduced on the TNSM and CP-Ti substrates (compared with expression on TCPS and Ti65-M2 MEA), which could cause lower cell migration and proliferation. Additionally, the lower expression of HIFα in U-87 MG on all Ti-containing alloys, compared with expression on TCPS, could lead to reduced angiogenesis. mTOR, eIF4, and S6 also show lower expression in cells grown on the alloys, Figure 6b, which could lower cell proliferation, growth, and translation. Figure 6c shows expressions of AKT, BAD, and GSK3, which may correlate with cell proliferation and survival. The lower expression of GSK3 could lower the cell proliferation and survival of U-87 MG cells on these Ti-containing materials. Finally, the gene expression of FOXO was low, reflecting low cell cycle arrest (Figure 6d). The higher expression of p53 and NFkβ in U-87 MG on CP-Ti, compared with cells on the other Ti alloys, showed that the CP-Ti may induce apoptosis. These results may explain the viability results for U-87 MG cells on the Ti alloys in Figure 4b.

## 3. Materials and Methods

### 3.1. Manufacture of Ti-Rich Medium-Entropy Alloy

Ti_65_-Zr_18_-Nb_16_-Mo_1_ (Ti65M), Ti-13Nb-7Sn-4Mo (TNSM), and CP-Ti were manufactured with an arc-melting and casting system (A-028, Dawn Shine, New Taipei City, Taiwan). After casting, Ti65M, TNSM, and CP-Ti were cut into a disc shape with a diameter of 10 mm and a height of 1 mm. The surfaces of all samples were abraded to #1500 silicon carbide papers, then cleaned using an ultrasonic cleaner (DC300, Delta, New Taipei City, Taiwan) with alcohol, acetone, and deionized water, successively.

### 3.2. Characterization of Ti-Rich Medium-Entropy Alloy

Phase identification was carried out with an X-ray diffractometer (XRD) (D8-Advance, Bruker, Germany) using the Cu-Kα radiation at 40 kV, 40 mA, 2θ = 20–90°, scanning speed = 4°/min, and step size = 0.02°/step. A Vickers hardness tester implemented a micro-hardness test (HMV-2T, Shimadzu, Kyoto city, Japan), using a previously described method [21]. The alloy’s water contact angle measurement was tested using the sessile drop method (1μL deionized water) using the contact angle meter (model 100SL, Sindatek, Taipei city, Taiwan). Three different areas on three different samples of each alloy will be measured, and the average value and standard deviation will be calculated. Surface topography and average roughness were performed by atomic force microscope (AFM) (Solver PRO-M, NT-MDT, Moscow, Russia) at an area scanning range of 5 × 5 μm^2^.

## 4. Conclusions

This study was performed to characterize the surfaces of novel medium-entropy alloy materials and to evaluate their biocompatibility with glioblast cells for potential medical applications. Material properties (phase structures, surface morphology, and roughness, and hydrophilicity) of Ti_65_-Zr_18_-Nb_16_-Mo_1_ (Ti65M), Ti-13Nb-7Sn-4Mo (TNSM), and CP-Ti were examined and are listed in Table 1. The biocompatibility, as assessed primarily by cell viability, and the expression of growth factor pathway mRNAs were studied. The cell viabilities on both Ti65M and TNSM (with β crystal structures) were considerably higher than that on CP-Ti (with an α’ crystal structure). The higher viabilities on Ti65M and TNSM also correlated with higher surface hardness and lower roughness. A study of growth factor pathways revealed that higher expressions of RAS, ERK, and S6 were associated with higher growth of U87MG cells on Ti65M MEA than on TNSM or CP-Ti, and could also lead to increased cell migration, a subject for further study. Cell growth on CP-Ti substrates was correlated with higher expression of p53 (compared with cells grown on the other alloys). P53 could induce apoptosis, and thus lower viability. In summary, this study showed that the Ti65M MEA, with a lower Ti concentration, was more biocompatible than the other materials containing higher Ti content. The good viability on lower Ti medium-entropy alloys indicates that these are good candidates for medical applications.

## Figures and Tables

**Table 1:** Material properties (phase structures, surface morphology and roughness, and hydrophilicity) of Ti_65_-Zr_18_-Nb_16_-Mo_1_ (Ti65M), Ti-13Nb-7Sn-4Mo (TNSM), and CP-Ti.

	Samples	Titanium-Containing Samples		
Properties		Ti_65_-Zr_18_-Nb_16_-Mo_1_ (Ti65M)	Ti-13Nb-7Sn-4Mo (TNSM)	Commercially Pure Titanium (CP-Ti)
Phase Structures		β	β	α’
Hardness (HV)		366.5 ± 3.6	235.1 ± 1.4	172.3 ± 4.4
Roughness (Sa, nm)		38 ± 8	43 ± 6	94 ± 26
Contac Angle (°)		54.3 ± 2.0	46.2 ± 2.7	46.0 ± 0.9
Viability (%)		97.9 ± 0.6	95.3 ± 2.6	91.1 ± 3.7

## Data Availability

The authors confirm that the data supporting the findings of this study are available within the article and its Supplementary Materials.

## Supplementary Materials

The following supporting information can be downloaded at: https://www.mdpi.com/article/10.3390/ijms232314552/s1, S1: Experimental; Table S1: The sequence (5’–3’) of primers for GAPDH, RAS, ERK, HIF1α, mTOR, eLF4, S6, AKT, GSK3, BAD, FOXO, p53, NFκβ; Scheme S1: Simplified schematic representation of RTK activation and the resultant downstream signaling; Figure S1: Optical and nuclear staining and their merge images of U-87 MG cells, which were incubated with TCPS (controls), Ti65M MEA, TNSM and CP-Ti for one day and transferred to TCPS culture [44,45].
