# Supplementary material for: Biocompatibility of a Ti-Rich Medium-Entropy Alloy with Glioblastoma Astrocytoma Cells
Source: Int J Mol Sci. 2022 Nov 22;23(23):14552. doi: 10.3390/ijms232314552 (PMC9741175; doi:10.3390/ijms232314552)
Supplement: Supplementary file 1 [file ijms-23-14552-s001.zip › ijms-2009899-supplementary.pdf]

Supporting inforamtion:

Biocompatibility of a Ti-rich Medium Entropy Alloy with Glioblastoma Astrocytoma Cells

S1. Experimental

S1.1 Cytotoxicity and Immunohistochemistry of U-87 MG Glioblastoma Astrocytoma Cells with Ti-rich Medium Entropy Alloy

The U-87 MG glioblastoma astrocytoma cell line was purchased from the Bioresource Collection and Research Center (#60360 BCRC; Taipei, Taiwan). Biocompatibility was tested in two different ways, following ISO 10993-1. First, the toxicity of any elements, compounds, or nanoparticles that could be extracted from the Ti alloys was studied. Traditional biocompatibility extraction methods were employed for Ti65-M2 MEA, TNSM and CP-Ti, and the extracts without additional processing were added to the Eagle's minimum essential medium (EMEM) medium in 10 vol% to the U-87 MG cells (1×10^4 cells) and incubated 24h before MTT measurements. Secondly, we studied cell directly growth on Ti substrates, and the U-87 MG cells (1×10^4 cells) were seeded onto the Ti_{65}–Zr_{18}–Nb_{16}–Mo_1 (Ti65M), Ti–13Nb–7Sn–4Mo (TNSM), CP–Ti samples (1.2 or 1.0 cm in diameter) in 24-well plates (tissue culture polystyrene, TCPS) in EMEM medium at 37 °C and 5% CO_2 for 24 h. After 24 h, the cells were trypsinized, gently washed and replated into a new 24-well plate (without titanium alloy samples) and grown for an additional 24 h prior to MTT tests. (This procedure was employed because 1) the MTT test requires measurement of absorption, and the opaque substrate would prevent this; and 2) direct fluorescence microscopy on the titanium alloy was not feasible.) (1) The MTT test was used for assessing the viability of cells after exposure; briefly, MTT solution (10 μL) was added to each well and incubated for 3 h, followed by removal of media and addition of 100 μL DMSO to dissolve the formazan crystals. Absorbance at wavelengths of 570 nm and 690 nm was measured with a plate reader (CLARIO star, BMG LABTECH, Germany). Cell viability (%) was calculated as a percentage with respect to untreated cells. (2) DAPI fluorescence. MEM was removed and then cells were washed with phosphate buffered saline (PBS) three times in each well. Cells were fixed with 3.7% paraformaldehyde (PFA) for 10 min. After removing PFA, cells were washed with PBS three times. 0.1% Triton-X-100/PBS was used to permeabilize cells for 5 minutes. This solution was removed, and then PBS was added and cells were washed three times. Bovine serum albumin in PBS at 7.5% was added and incubated at 4 ºC for 1 h. The

bovine serum albumin solution was removed, and then washed with PBS three times. DAPI at 1 μL/mL in PBS was added to stain cells for 5 minutes. PBS was added and cells washed three times after DAPI was removed. The 24-well culture plate was placed in the dark at room temperature until each well was dry. The morphologies of cell nuclei were obtained using an inverted fluorescence microscope (CKX41, Olympus, Melville, NY).

Electron microscopy of U-87 MG glioblastoma astrocytoma cell line on samples were immersed in (1) 2.5 % gluteradehyde at 4 °C for 2 h; (2) 30, 50, 70, 80, 90 and 100 % ethanol solutions for 10 to 15 min; (3) acetone 15 min, twice, for dehydration. Samples were freeze-dried overnight and coated with Au and then examined in the electron microscope.

S1.2 Gene expression of U-87 MG Glioblastoma Astrocytoma Cells with Ti-rich Medium Entropy Alloy

The sequence (5'- 3') of primers for GAPDH, RAS, ERK, HIF1α, mTOR, eLF4, S6, AKT, GSK3, BAD, FOXO, p53, and NFκβ are listed in Table S1. Glioblastoma astrocytoma cells were centrifuged at 1000 rpm for 5 min to remove culture medium. The total RNA extraction from U87-MG cells on T65M, TNSM, and CP–Ti samples was accomplished using the Nucleospin RNA, Mini kit for RNA purification (740955.50 Macherey-Nagel). Complementary DNA was obtained following a Magic RT Mastermix cDNA synthesis kit (BB-DBU-RT-100, Bio-genesis Technologies, lnc., Taiwan). Real-time PCR was then performed with IQ2 SYBR Green Fast qPCR System Master Mix (BB-DBU-006-5, Bio-genesis Technologies, lnc., Taiwan) in a StepOne™ Real-Time PCR System (LS4376357, Applied Biosystems, Waltham, MA). Relative gene expression was determined using a ΔΔCq method [1] and normalized to a reference gene (GAPDH) and to control (glioblastoma astrocytoma cells).

S1.3 Data Analysis

All experiments were carried out in triplicate, and data are expressed as means ± standard deviation. The gene expression data were analyzed with Student's t-test. Statistical significance (*) was set at a p-value of less 0.05, and highly significant (**) as $p<0.001$.

Reference:

1. Pfaffl, M.W. A new mathematical model for relative quantification in real-time RT-PCR. *Nucleic Acids Res* **2001**, *29*, e45-e45, doi:10.1093/nar/29.9.e45.
2. Pearson, J.R.D.; Regad, T. Targeting cellular pathways in glioblastoma multiforme. *Signal Transduction and Targeted Therapy* **2017**, *2*, 17040, doi:10.1038/sigtrans.2017.40.

Table S1. The sequence (5'–3') of primers for GAPDH, RAS, ERK, HIF1α, mTOR, eLF4, S6, AKT, GSK3, BAD, FOXO, p53, NFκβ.

mRNA	Forward/Reverse	Sequence (5'–3')
GAPDH	forward	ACAGTTGCCATGTAGACC
	reverse	TTGAGCACAGGGTACTTTA
RAS	forward	AATGCAGGATCAAGAACAAG
	reverse	AAGGCATCCTTTGTTTTACG
ERK	forward	TTCGAACATCAGACCTACTG
	reverse	TAGACATCTCTCATGGCTTC
HIF1α	forward	AAAATCTCATCCAAGAAGCC
	reverse	AATGTTCCAATTCCTACTGC
mTOR	forward	GGAGGAGAAATTTGATCAGG
	reverse	GGGCAACAAATTAACGATTG
eIF4	forward	CCTATTTTTAGTGGTGGAGC
	reverse	CATATAGGGAGGGTATACAAGG
S6	forward	GGATATACTCCATTTGCCAAC
	reverse	AAACTGTGTTCCAATTTCC
AKT	forward	AAGTACTCTTTCCAGACCC
	reverse	TTCTCCAGCTTGAGGTC
GSK3	forward	CAAATGTCTCCTACATCTGTTC
	reverse	TCGATGGATGAGGTGTAATC
BAD	forward	ATCATGGAGGCGCTG
	reverse	CTTAAAGGAGTCCACAAACTC
FOXO	forward	GTCAAGACAACGACACATAG
	reverse	AAACTAAAAGGGAGTTGGTC
p53	forward	AGGATTCACAGTCGGATATG
	reverse	GGAGGAAGAAGTTTCCATAAG
NFκβ	forward	CACAAGGAGACATGAAACAG
	reverse	CCCAGAGACCTGATAGTTG

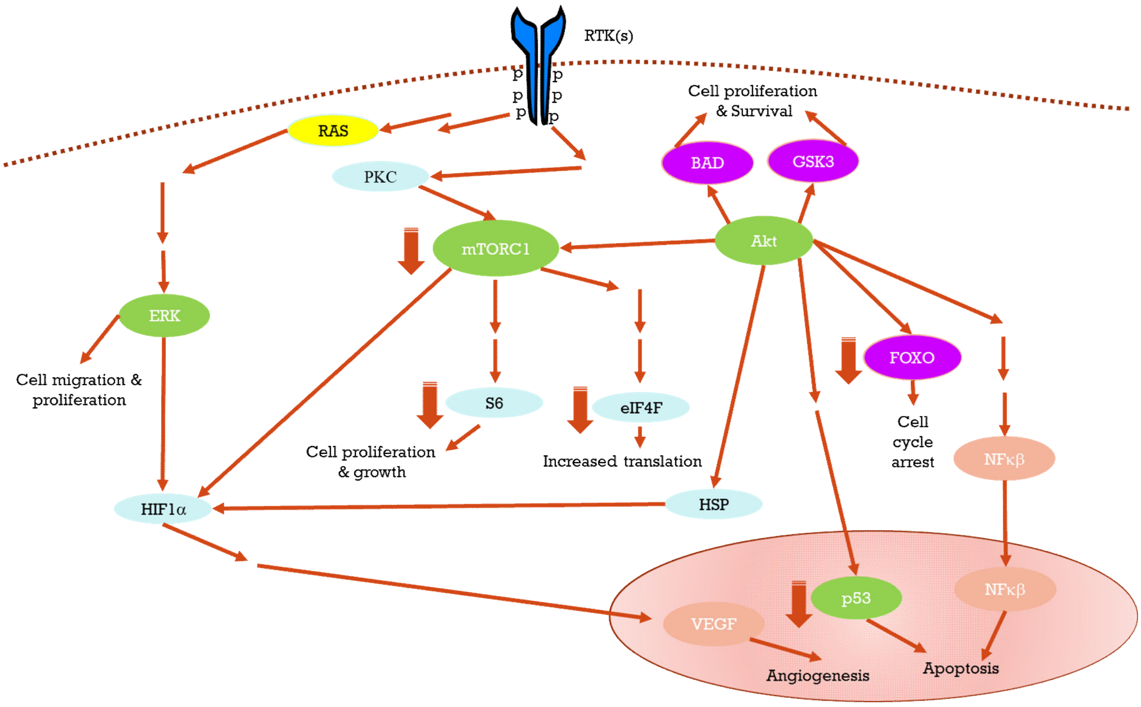

Scheme S1. Simplified schematic representation of RTK activation and the resultant downstream signaling [2].

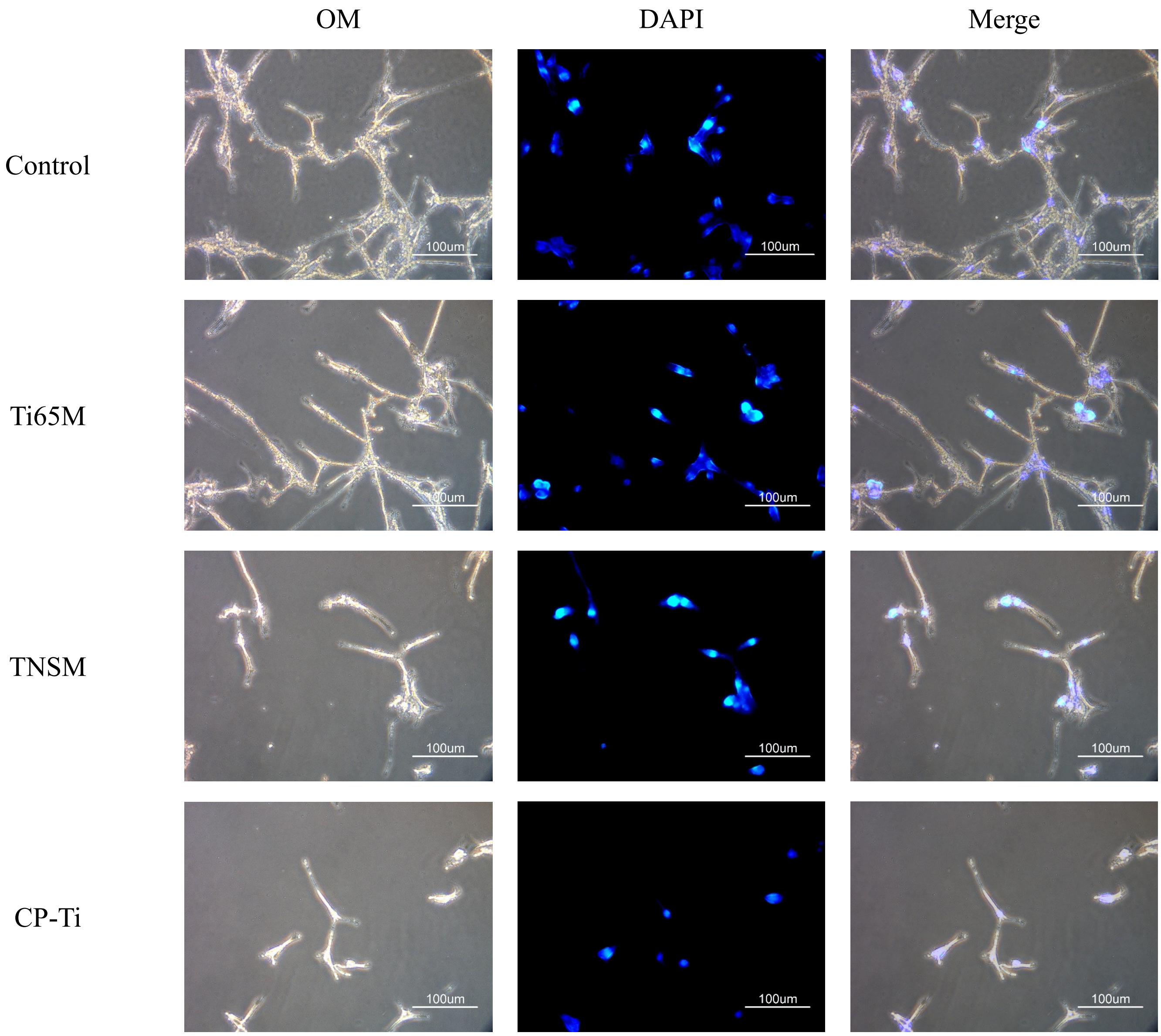

Figure S1. Optical and nuclear staining and their merge images of U-87 MG cells, which were incubated with TCPS (controls), Ti65M MEA, TNSM and CP-Ti for one day and transferred to TCPS culture.
